# Adherence to physical activity recommendations and associations with self-efficacy among Norwegian adolescents: trends from 2017 to 2021

**DOI:** 10.3389/fpubh.2024.1382028

**Published:** 2024-05-23

**Authors:** Erik Grasaas, Øyvind Sandbakk

**Affiliations:** ^1^Teacher Education Unit, University of Agder, Kristiansand, Norway; ^2^Department of Neuromedicine and Movement Science, Center for Elite Sports Research, Norwegian University of Science and Technology, Trondheim, Norway; ^3^School of Sport Science, UiT The Artic University of Norway, Tromsø, Norway

**Keywords:** exercise habits, health behavior, mental health, PA recommendations, adolescents

## Abstract

**Background:**

The crucial role of physical activity (PA) in promoting well-being and overall health of adolescents is widely acknowledged. Previous global studies have consistently revealed low adherence to PA recommendations among adolescents, emphasizing potential links between PA engagement and self-efficacy in school-based populations. However, there is a need for further exploration of this relationship, in particularly gender differences and taking into account the potential influences of the COVID-19 pandemic. The objective of this study is to provide a comprehensive description of adherence to PA recommendations and its associations with self-efficacy in Norwegian school-based adolescents over the period from 2017 to 2021.

**Methods:**

Cross-sectional data on physical activity (PA) levels and self-efficacy among 13–19-year-old Norwegian adolescents were collected from the Norwegian Ungdata Survey during the period 2017 to 2021. The survey, conducted in Norwegian lower- and upper-secondary schools, was administered electronically during school hours. All data collected is anonymous and has received approval from the Norwegian Agency for Shared Services in Education and Research (SIKT). Statistical analyses were performed using SPSS software.

**Results:**

Girls consistently exhibited lower adherence to PA recommendations (17.6–19.8%) compared to boys (27.7–31.1%) each year from 2017 to 2021 (all *p* < 0.01). Similarly, girls reported lower self-efficacy (14.1 to 14.8 out of 20) than boys (15.5 to 15.9) during the same period (all *p* < 0.01). Regression analyses highlighted robust positive associations between PA and self-efficacy in those adhering to PA recommendations (i.e., physically active at least 5 times a week) and strong inverse associations for those reporting inactivity (never active) in both girls and boys from 2017 to 2021.

**Conclusion:**

Adolescents in Norway report PA adherence ranging from 15 to 30%, with girls consistently exhibiting lower adherence to PA recommendations and reporting lower self-efficacy than boys. Notably, there are substantial associations between self-efficacy and both adherence to PA recommendations and inactivity over time. These findings underscore the significance of promoting adherence to PA recommendations during adolescence, especially among girls. Policymakers in Norway should focus on initiatives to increase PA levels among adolescents in both lower and upper secondary schools.

## 1 Background

Physical activity (PA) is crucial for the well-being and overall health of adolescents (1). Adolescence represents a critical period encompassing physical, psychological, and social development, with a pronounced emphasis on the pursuit of independence (1–3). During this phase, behavioral habits are often formed, and the decision to engage in physical activity emerges as a significant determinant of future health (1, 4).

Extensive evidence underscores physical inactivity in adolescence as a predictor of increased risks for developing non-communicable chronic diseases, elevated morbidity and mortality rates, as well as heightened susceptibility to economic burdens on society due to social support requirements and diminished work capacity (5–7). Consequently, there is a growing global concern about inactivity, and international data reveals that 81% of adolescents fail to meet PA guidelines, with substantial variations observed across countries, regions, genders, and religions (8). A comprehensive study involving 146 countries disclosed that 84.7% of girls and 77.6% of boys did not achieve sufficient physical activity levels (8). Harmonizing accelerometer measures of PA across Europe, Steen-Johannsen and colleagues demonstrated that two-thirds of European children and adolescents are inadequately physically active (9). Furthermore, the findings suggested that boys exhibited higher activity levels and lower sedentary behavior across all age categories compared to girls. Both girls and boys demonstrated a yearly reduction in PA levels from 13 to 17 years of age (9).

For children and adolescents aged 5 to 17 years, the World Health Organization (WHO) recommends “*at least an average of 60 min per day of moderate-to-vigorous intensity, mostly aerobic, physical activity, across the week and should incorporate vigorous-intensity aerobic activities, as well as those that strengthen muscle and bone, at least 3 days a week*.” According to the Norwegian public health report from 2018 (Folkehelserapporten), half of the boys and 40% of girls aged 15 adhered to the PA guidelines in Norway (10, 11). Given the emergence of Covid-19, which has significantly impacted the daily lives of school-based adolescents, there is a clear need for updated research on adherence to PA recommendations over time, both before and after the pandemic, in Norwegian girls and boys.

According to the systematic review and meta-analysis by Rodriguez-Ayllon and colleagues, investigating the role of PA and sedentary behavior on the mental health of preschoolers, children, and adolescents (12), revealed that PA, especially among adolescents, can improve their self-efficacy. The theory of self-efficacy focuses on how performance or behavior influences one’s beliefs (13) and according to Albert Bandura, self-efficacy is defined as “*how well one can execute courses of action required to deal with prospective situations*” (14). Self-efficacy has also been shown to be a predictor for PA, presumably as a motivator and self-regulating mechanism (15, 16). However, the relationship is complex because self-efficacy is argued to influence PA engagement and be influenced by PA engagement (17).

Self-efficacy has been identified as an important determinant of both present and future health behavior (18–21). Bandura argues that self-efficacy impacts activity choices, persistence when facing challenges or barriers, and the effort or intensity in tasks (22). A higher belief in one’s own capacity impacts activity choices and activity level. As a result of a higher PA level, there is even higher self-efficacy, thereby creating a positive feedback loop. Thus, engagement in PA seems to be a preferred method for increasing self-efficacy, as engaging in PA boosts the feeling of success and self-belief (23, 24).

According to Spence and colleagues, self-efficacy also contributes to explain gender differences in PA levels among adolescents, in which boys are shown to have higher self-efficacy compared with girls (25). Norwegian studies have also reported that girls tend to report lower self-efficacy than boys in both younger and older adolescents (26–28). Previous findings have revealed a significant differences in self-efficacy of young people doing exercise regularly compared to sedentary ones (23). Further, research evidence indicates that promoting different types of physical activities provide an increase in self-efficacy in school-based adolescents, such as yoga interventions and resistance training (29, 30). Hence, PA engagement might be a relevant indicator for the degree of self-efficacy in Norwegian school-based adolescents regardless of activity type, yet this remains unknown.

There is a clear need to investigate PA levels and self-efficacy in a school-based sample of Norwegian adolescents before and after the pandemic using nationwide data. As the concept of self-efficacy is interwoven with PA and is reported to be a self-regulatory mechanism by which change is possible (31, 32), it is particularly interesting to address these relationships over time. By using nationwide data over time to explore how different levels of PA impact self-efficacy in Norwegian girls and boys, a clearer link between PA levels and mental health benefits can be established. Moreover, such insights may enhance our understanding of how much PA is required to impact the directional nature of self-efficacy. Therefore, the objective of this study is to provide a comprehensive description of adherence to PA recommendations and its associations with self-efficacy in Norwegian school-based adolescents over the period from 2017 to 2021.

We hypothesize that: (i) PA levels and self-efficacy are lower in girls than boys. (ii) Adherence to PA recommendations (highest level of PA) is strongly associated with self-efficacy in girls and boys every year from 2017–2021. (iii) Inactivity (lowest level of PA) is inversely associated with self-efficacy in both girls and boys.

## 2 Methods

### 2.1 Study design and participants

This study utilized cross-sectional data from the Norwegian Ungdata Survey, conducted annually from 2017 to 2021. Ungdata is a nationwide survey recognized as the most comprehensive source of information on Norwegian adolescents’ health and lifestyle (33).

The study includes Norwegian adolescents from lower (aged 13 to 16 years of age) and upper secondary school (aged 16 to 19 years of age). The national reports from Ungdata encompass data from the last three recent years. Between 2017 and 2019, a total of 259,700 adolescents participated, with 146,400 responders from lower secondary school (grades 8th, 9th, and 10th) and 113,300 responders from upper secondary school (grades 1st, 2nd, and 3rd). During this period, 80% of all lower secondary school pupils and 60% of all upper secondary school pupils in Norway took part. From 2018 to 2020, the participation rates were 79% in lower secondary school and 65% in upper secondary school. Notably, due to the pandemic, findings from 2021 are reported separately. In 2021, a total of 140,000 pupils from 8th grade to 3rd grade participated, reflecting an 83% response rate from lower secondary schools and a 67% response rate from upper secondary school (34).

### 2.2 Outcomes

The Ungdata study encompasses demographic measures, including gender, grade level, respective municipalities, and various health-related questions. Due to the survey’s anonymity, age data is not available.

Physical activity (PA) levels were assessed using the question, “*How often are you so physically active that you become short of breath or sweaty?*” Respondents could choose from six response alternatives ranging from “never active” to different times a week, up to “at least 5 times a week.” In this study, the response “at least 5 times a week” was used as a proxy for compliance with World Health Organization (WHO) recommendations for PA. Single-item measures of PA have demonstrated strong reliability and concurrent validity (35). The single-item measure of PA is considered a potentially useful assessment tool for evaluating changes in moderate-vigorous PA levels, especially when device-based measures or longer questionnaires are impractical (36). Given the study period spanning from 2017 to 2021, the applied physical activity (PA) questions were considered most appropriate for addressing the current paper’s aim. However, enhancing the single-item measure, such as incorporating more comprehensive or objective measures, would have improved the sensitivity to PA levels in subjects. This PA question was part of the mandatory module of the Ungdata survey, included in all participating municipalities.

Self-efficacy was measured using the Norwegian 5-item version of the General Perceived Self-Efficacy Scale (GSE) (37). GSE is developed for assessing the global confidence in one’s abilities to cope with the tasks, demands, and challenges of life in general and reported as a valid and reliable psychometric scale (38–40). GSE encompass five statements, rated on a scale from 1 (completely wrong) to 4 (completely right). Scores of the GSE items are summed into a total score ranging from 5 to 20, wherein higher scores indicate higher GSE levels. Questions related to self-efficacy were part of the optional module, and thus, the inclusion of these questions varied across municipalities from 2017 to 2021 (Supplementary File S1).

Covariates considered in the adjusted analysis for each year (2017–2021) included the following factors: socioeconomic status (SES), perceived school stress as an indicator of psychological well-being (41), and over-the-counter analgesics (OTCA) use as an indicator of health status. SES was assessed using several questions related to parental educational level, the presence of books in the home, and the level of prosperity. The total sum of these three categories was calculated and recoded into values ranging from 0 to 3, with 0 representing the lowest SES and 3 the highest SES (42). This measure is reported as a validated construct of SES (42). Perceived school stress was measured by the statement “*I get stressed by the schoolwork?”* with five response alternatives: “never,” “seldom,” “sometimes,” “often” and “very often.” OTCA use was assessed with the question *“How often have you used non-prescription drugs (Paracet, Ibux and similar) during the last month?”* Participants could choose from five response alternatives: “never,” “less than once a week,” “at least weekly,” “several times a week,” and “daily.”

### 2.3 Data collection

Ungdata is conducted by Norwegian Social Research (NOVA) at Oslo Metropolitan University in collaboration with the regional center for drug rehabilitation (KoRus) and the municipal sector’s organization (KS). Surveys are administered electronically during one school hour with a teacher present. Pupils who choose not to participate are provided with alternative schoolwork assignments. The survey comprises a mandatory basic module for all municipalities and some optional questions that municipalities can select from. Adolescents from nearly all municipalities in Norway are represented, with different municipalities participating each year. According to Ungdata, the research evidence derived from these surveys is well-suited for planning and initiating interventions related to adolescents and public health (33). The Ungdata project is financed from the national budget through grants from the Norwegian Directorate of Health (33).

### 2.4 Ethical consideration

Participation in the Ungdata survey is voluntary, and informed written consent was obtained from the adolescents. All questions from Ungdata included in this current study have been approved by the Norwegian Agency for Shared Services in Education and Research (ref. 821,474), known as SIKT (43). As the survey is conducted during the spring, adolescents in upper secondary school were 16 years or older and thus did not need parental consent. Participants in lower secondary school required additional parental approval to participate. Due to the anonymity of the data, age was not included. The study is conducted in accordance with the Helsinki Declaration and is reported according to the Strengthening the Reporting of Observational Studies in Epidemiology (STROBE) guidelines (44).

### 2.5 Statistical analyses

All statistical analyses were performed using IBM SPSS Statistics for Windows, Version 25.0 (IBM Corp., Armonk, NY, USA). Descriptive measures for continuous variables are presented as means and standard deviations (SDs), while categorical variables are reported as counts and percentages. Adherence to PA levels was categorized into those likely complying with WHO guidelines for PA recommendation (reporting PA at least 5 times a week) and those not complying (all other categories). Descriptive measures were stratified by grade levels (8th to 3rd grade) and by gender. Chi-square tests and t-tests were employed to assess yearly differences between boys and girls for PA levels and self-efficacy, respectively.

Linear regression analyses were conducted to explore the association between PA levels and self-efficacy. The predicting independent categories of PA were recoded into dummy variables. Both crude and adjusted multiple regression analyses were presented, with regressions displaying beta coefficients along with 95% confidence intervals. The adjusted regression analysis included covariates such as SES, perceived school stress, and OTCA use. *p*-values <0.05 were considered statistically significant, and all tests were two-sided. Given the large sample size and low missing, bootstrapping nor imputation was not considered necessary.

## 3 Results

### 3.1 Participants

A total of 433,046 Norwegian school-based adolescents reported their PA levels, and 196,786 reported their self-efficacy between 2017 and 2021. Among them, 195,557 adolescents provided responses for both measures. The response rate for PA levels remained consistently high, ranging from 91.9 to 93.8% each year, while the response rate for self-efficacy varied more (from 51.8 to 77.4%) and was not consistently reported across all municipalities (refer to Supplementary File S1 for details). An equal gender distribution was observed throughout the period, ranging from 49.6 to 50.4%.

### 3.2 Descriptive statistics

The total scores revealed lower adherence to PA recommendations in girls compared to boys every year from 2017 to 2021 (*p* < 0.01). Adherence to PA recommendations for girls ranged from 17.6 to 19.8% during the same period (Table 1). Girls reported lower adherence to PA recommendations in upper secondary school (1st – 3rd grade) compared to lower secondary school (8th to 10th grade) every year. There was a tendency of decreasing adherence to PA levels over time as the girls’ cohorts progressed through yearly grading levels, with the lowest adherence typically found in the 3rd year, except for 2020.

**Table 1:** Overview of adherence to physical activity recommendations (expressed as a percentage) in Norwegian girls and boys by grade level from 2017 to 2021.

	Grade Level						
	8th grade	9th grade	10th grade	1st year	2nd year	3rd year	Total score
Year: 2017							
Girls	23.3%	22.5%	20.8%	17.7%	16.7%	13.5%	19.8% *
	*n* = 9,443	*n* = 8,963	*n* = 8,988	*n* = 8,579	*n* = 6,053	*n* = 4,385	*n* = 47,552
Boys	32.4%	33.7%	32.2%	29.5%	28.4%	27.9%	31.1%
	*n* = 9,570	*n* = 9,072	*n* = 9,010	*n* = 8,604	*n* = 5,806	*n* = 3,376	*n* = 46,691
Year: 2018							
Girls	20.2%	20.8%	18.9%	15.6%	14.7%	13.0%	17.6% *
	*n* = 6,349	*n* = 6,278	*n* = 5,909	*n* = 5,710	*n* = 4,712	*n* = 4,034	*n* = 33,668
Boys	29.2%	28.8%	30.1%	26.4%	23.5%	26.9%	27.7%
	*n* = 6,154	*n* = 6,083	*n* = 5,627	*n* = 5,461	*n* = 4,622	*n* = 3,032	*n* = 31,668
Year: 2019							
Girls	21.8%	21.4%	20.4%	17.7%	16.5%	13.5%	18.8% *
	*n* = 10,112	*n* = 9,826	*n* = 9,821	*n* = 10,595	*n* = 9,105	*n* = 5,918	*n* = 56,337
Boys	29.1%	30.4%	31.9%	27.4%	26.7%	28.3%	28.9%
	*n* = 9,898	*n* = 9,606	*n* = 9,502	*n* = 10,474	*n* = 9,183	*n* = 4,698	*n* = 54,704
Year: 2020							
Girls	20.2%	20.8%	19.9%	19.3%	16.7%	16.9%	19.0% *
	*n* = 2,114	*n* = 1966	*n* = 2079	*n* = 2,302	*n* = 1806	*n* = 1771	*n* = 12,403
Boys	29.9%	32.9%	34.0%	29.7%	29.3%	32.5%	31.1%
	*n* = 2,116	*n* = 1850	*n* = 2,127	*n* = 2,264	*n* = 2,528	*n* = 1,097	*n* = 11,731
Year: 2021							
Girls	19.3%	19.9%	20.5%	17.7%	17.1%	16.7%	18.7% *
	*n* = 12,455	*n* = 12,401	*n* = 12,341	*n* = 10,597	*n* = 9,067	*n* = 7,146	*n* = 65,495
Boys	29.7%	32.6%	34.2%	29.4%	28.3%	31.9%	31.0%
	*n* = 12,459	*n* = 12,425	*n* = 12,021	*n* = 10,594	*n* = 8,635	*n* = 4,743	*n* = 62,423

n, the number of total responders, %, the percentage of the total responders adhering to the recommendation of physical activity (reported at least 5 times).* = *p* < 0.01.

For boys, adherence to PA recommendations ranged from 27.7 to 31.1% from 2017 to 2021. Boys reported relatively consistent adherence to PA recommendations from 8th grade to 3rd year during the same period. The highest yearly adherence was found in either 9th or 10th grade (33.7% or 34.2%, respectively). The lowest levels of adherence were found in the 2nd year for boys in most years, except for 2017.

The total score indicates that girls consistently reported lower self-efficacy (ranging from 14.1 to 14.8 out of 20) than boys (15.5 to 15.9) each year from 2017 to 2021 (all years *p* < 0.01). The findings revealed minimal to negligible fluctuations in reported self-efficacy from 8th grade to 3rd year annually (Table 2). Girls demonstrated a gradual decrease in the total score of self-efficacy each year, declining from a mean (SD) of 14.8 (2.7) in 2017 to 14.1 (2.9) in 2021. Additionally, the results unveiled slight variations in reported self-efficacy among boys from 8th grade to 3rd year, with the highest self-efficacy score consistently observed in the 3rd year for boys each year in the period. The mean total score for boys was marginally higher in 2017 (mean (SD): 15.9 (2.8)) compared to 2021 (mean (SD): 15.5 (3.0)), yet no clear trend was identified.

**Table 2:** Overview of self-efficacy (mean/SD) in Norwegian girls and boys by grade level from 2017 to 2021.

	Grade level						
	8th grade	9th grade	10th grade	1st year	2nd year	3rd year	Total score
Year: 2017							
Girls	15.0 (2.7)	14.6 (2.7)	14.6 (2.7)	14.7 (2.7)	14.8 (2.7)	14.9 (2.5)	14.8 (2.7) *
	*n* = 2,872	*n* = 2,867	*n* = 2,909	*n* = 3,693	*n* = 3,171	*n* = 2,543	*n* = 18,222
Boys	15.9 (2.8)	15.7 (2.8)	15.8 (2.9)	16.0 (2.8)	16.0 (2.8)	16.2 (2.8)	15.9 (2.8)
	*n* = 2,950	*n* = 2,869	*n* = 2,793	*n* = 3,569	*n* = 3,085	*n* = 1967	*n* = 17,416
Year: 2018							
Girls	14.6 (3.1)	14.3 (2.9)	14.5 (2.9)	14.5 (2.8)	14.6 (2.9)	14.5 (2.5)	14.5 (2.8) *
	*n* = 319	*n* = 376	*n* = 323	*n* = 849	*n* = 673	*n* = 611	*n* = 3,201
Boys	15.2 (3.3)	15.3 (3.3)	15.7 (3.2)	15.5 (3.3)	15.5 (3.1)	16.0 (2.7)	15.5 (3.2)
	*n* = 304	*n* = 370	*n* = 328	*n* = 796	*n* = 727	*n* = 429	*n* = 3,017
Year: 2019							
Girls	14.6 (3.1)	14.4 (3.0)	14.5 (2.9)	14.6 (2.9)	14.7 (2.8)	14.8 (2.6)	14.6 (2.9) *
	*n* = 7,520	*n* = 7,696	*n* = 7,851	*n* = 8,041	*n* = 7,157	*n* = 4,557	*n* = 43,290
Boys	15.5 (3.1)	15.5 (3.2)	15.6 (3.1)	15.8 (3.2)	15.9 (3.0)	16.1 (2.9)	15.7 (3.1)
	*n* = 7,388	*n* = 7,399	*n* = 7,506	*n* = 8,114	*n* = 7,186	*n* = 3,715	*n* = 41,799
Year: 2020							
Girls	14.5 (2.8)	14.2 (3.0)	14.4 (2.8)	14.4 (2.7)	14.5 (2.5)	14.9 (2.7)	14.5 (2.8) *
	*n* = 999	*n* = 922	*n* = 1,084	*n* = 1,249	*n* = 1,025	*n* = 1,101	*n* = 6,572
Boys	15.6 (3.0)	15.5 (3.0)	15.7 (2.9)	15.5 (3.0)	15.7 (3.0)	16.0 (2.8)	15.7 (3.0)
	*n* = 972	*n* = 901	*n* = 1,064	*n* = 1,188	*n* = 1,005	*n* = 664	*n* = 5,971
Year: 2021							
Girls	13.9 (3.1)	13.9 (3.0)	14.1 (2.9)	14.2 (2.9)	14.3 (2.8)	14.5 (2.7)	14.1 (2.9) *
	*n* = 4,313	*n* = 4,628	*n* = 4,701	*n* = 5,146	*n* = 4,392	*n* = 3,584	*n* = 27,465
Boys	15.2 (3.1)	15.2 (3.1)	15.4 (3.1)	15.6 (3.0)	15.8 (3.0)	15.9 (2.9)	15.5 (3.0)
	*n* = 4,297	*n* = 4,709	*n* = 4,623	*n* = 4,986	*n* = 4,232	*n* = 2,349	*n* = 25,941

* = *p* < 0.01.

### 3.3 Regressions analyses

For both girls and boys, including crude and adjusted regressions, the findings consistently revealed the strongest positive associations among those adhering to PA recommendations (at least 5 times a week) and the strongest inverse (negative) associations for those reporting inactivity (never active) concerning self-efficacy (Table 3). This examination was conducted every year from 2017 to 2021. In the case of girls, those who did not meet PA recommendations exhibited a significantly inverse association (*p* < 0.01) with self-efficacy from 2017 to 2020. The association in 2021 was borderline significant (*B* = −0.81, 95% CI; [−1.66 to 0.04]) after adjusting for SES, perceived stress, and OTCA use (Table 3). After adjusting for selected covariates, girls reporting adherence to PA levels (at least 5 times a week) revealed a positive significant association (*p* < 0.05) with self-efficacy in 2018, 2020, and 2021. For boys, associations between adhering to PA recommendations (at least 5 times a week) and self-efficacy remained significant (*p* < 0.05) every year after adjusting for covariates from 2017 to 2021. Boys reporting inactivity showed an inversely significant association (*p* < 0.01) with self-efficacy every year, except in 2020.

**Table 3:** Association between physical activity levels and self-efficacy in Norwegian adolescents (2017–2021): gender and grade-level stratification in crude and adjusted analyses.

	Girls						Boys					
	Unadjusted			Adjusted			Unadjusted			Adjusted		
	*B*	95% CI	*p*-value	*B*	95% CI	*p*-value	*B*	95% CI	*p*-value	*B*	95% CI	*p*-value
2017												
Never	-1.59	-2.06: -1.13	<0.01	-1.41	-1.87: -0.95	<0.01	-1.67	-2.12: -1.22	<0.01	-1.56	-2.01: -1.11	<0.01
Seldom	-1.24	-1.57: -0.92	<0.01	-1.14	-1.46: -0.83	<0.01	-1.02	-1.34: -0.70	<0.01	-0.98	-1.31: -0.66	<0.01
1-2 times a month	-0.87	-1.19: -0.55	<0.01	-0.79	-1.10: -0.47	<0.01	-0.71	-1.03: -0.39	<0.01	-0.68	-1.00: -0.35	<0.01
1-2 times a week	-0.56	-0.86: -0.26	<0.01	-0.54	-0.83: -0.24	<0.01	-0.36	-0.63: -0.08	0.01	-0.42	-0.70: -0.14	<0.01
3-4 times a week	-0.24	-0.54: 0.05	0.11	-0.30	-0.60: -0.01	0.04	0.08	-0.19: 0.36	0.54	-0.00	-0.28: 0.27	0.99
At least 5 times	0.19	-0.11: 0.50	0.21	0.11	-0.19: 0.41	0.47	0.58	0.30: 0.85	<0.01	0.48	0.20: 0.75	<0.01
2018												
Never	-2.27	-3.38: -0.96	<0.01	-3.38: -0.96	-3.13: -0.72	<0.01	-1.88	-3.02: -0.74	<0.01	-1.74	-2.89: -0.58	<0.01
Seldom	-0.77	-1.58: 0.03	0.06	-1.58: 0.03	-1.41: 0.21	0.15	0.61	-0.23: 1.46	0.16	0.52	-0.34: 1.38	0.24
1-2 times a month	-0.25	-1.07: 0.57	0.55	-1.07: 0.57	-0.91: 0.74	0.84	0.69	-0.17: 1.54	0.11	0.66	-0.20: 1.53	0.13
1-2 times a week	-0.06	-0.82: 0.70	0.88	-0.82: 0.70	-0.73: 0.79	0.94	1.08	0.33: 1.82	<0.01	0.98	0.22: 1.75	0.01
3-4 times a week	0.37	-0.39: 1.13	0.34	-0.39: 1.13	-0.36: 1.17	0.30	1.41	0.66: 2.26	<0.01	1.31	0.55: 2.08	<0.01
At least 5 times	0.81	0.03:1.59	0.04	0.03:1.59	-0.00: 1.55	0.05	1.99	1.24: 2.74	<0.01	1.89	1.12: 2.67	<0.01
2019												
Never	-1.54	-1.97: -1.12	<0.01	-1.30	-1.73: -0.87	<0.01	0.98	-1.38: -0.58	<0.01	-1.12	-1.55: -0.68	<0.01
Seldom	-0.90	-1.28: -0.53	<0.01	-0.69	-1.08: -0.31	<0.01	-0.22	-0.57: 0.14	0.23	-0.35	-0.74: 0.04	0.08
1-2 times a month	-0.55	-0.93: -0.17	<0.01	-0.33	-0.72: 0.06	0.10	-0.06	-0.42: 0.29	0.74	-0.21	-0.60: 0.18	0.30
1-2 times a week	-0.18	-0.55: 0.19	0.34	-0.04	-0.41: 0.34	0.85	0.47	0.13: 0.80	<0.01	0.30	-0.08: 0.68	0.12
3-4 times a week	0.19	-0.18: 0.56	0.30	0.27	-0.11: 0.65	0.16	0.92	0.59: 1.26	<0.01	0.71	0.33: 1.08	<0.01
At least 5 time	0.62	0.25: 0.99	<0.01	0.67	0.29: 1.05	<0.01	1.49	1.15: 1.83	<0.01	1.26	0.89: 1.64	<0.01
2020												
Never	-0.98	2.12: 0.16	0.09	-1.68	-2.89: -0.48	<0.01	-0.51	-1.94: 0.93	0.49	-0.98	-2.57: 0.62	0.23
Seldom	-0.66	-1.69: 0.38	0.21	-1.14	-2.25: -0.03	0.04	0.78	-0.56: 2.12	0.26	0.10	-1.42: 1.62	0.90
1-2 times a month	-0.03	-1.06: 1.01	0.96	-0.57	-1.68: 0.53	0.31	0.84	-0.52: 2.19	0.23	0.27	-1.25: 1.80	0.73
1-2 times a week	0.11	-0.90: 1.12	0.83	-0.48	-1.57: 0.60	0.38	1.18	-0.14: 2.50	0.08	0.55	-0.95: 2.05	0.47
3-4 times a week	0.59	-0.42: 1.60	0.25	-0.09	-1.18: 0.99	0.87	1.63	0.32: 2.95	0.02	0.97	-0.53: 2.47	0.20
At least 5 times	1.18	0.16: 2.19	0.02	0.42	-0.67: 1.51	0.45	2.20	0.88: 3.52	<0.01	1.51	0.01: 3.01	0.05
2021												
Never	-0.64	-1.42: 0.13	0.10	-0.81	-1.66: 0.04	0.06	-1.66	-2.46: -0.86	<0.01	-1.40	-2.34: -0.46	<0.01
Seldom	-0.22	-0.96: 0.52	0.56	-0.37	-1.19: 0.45	0.37	-0.42	-1.19: 0.35	0.28	-0.22	-1.13: 0.69	0.64
1-2 times a month	0.46	-0.28: 1.20	0.22	0.26	-0.56: 1.08	0.53	-0.22	-0.99: 0.55	0.58	0.09	-0.83: 1.00	0.85
1-2 times a week	0.76	0.03: 1.49	0.04	0.50	-0.32: 1.30	0.23	0.34	-0.42: 1.10	0.38	0.50	-0.40: 1.40	0.28
3-4 times a week	1.12	0.39: 1.85	<0.01	0.80	-0.01: 1.61	0.05	0.78	0.03: 1.54	0.04	0.92	0.02: 1.82	0.05
At least 5 times	1.67	0.94: 2.40	<0.01	1.27	0.46: 2.08	<0.01	1.38	0.63: 2.14	<0.01	1.50	0.60: 2.40	<0.01

^Adjusted for: SES, perceived stress from school and over the counter analgesics use.

## 4 Discussion

This study aimed to investigate adherence to physical activity (PA) recommendations and its association with self-efficacy among Norwegian school-based adolescents from 2017 to 2021. Results consistently indicated lower adherence to PA recommendations and lower self-efficacy in girls compared to boys each year during the study period. Regression analyses highlighted robust positive associations between PA adherence (engaging in physical activity at least 5 times a week) and self-efficacy, while the strongest inverse associations were observed in those reporting inactivity (never active). These patterns held true for both girls and boys across all years from 2017 to 2021.

As hypothesized, our study consistently found lower adherence to PA recommendations in girls compared to boys each year, aligning with a global trend widely reported in the literature (8, 45–47). Our results closely parallel the international data presented by Guthold et al., who conducted a comprehensive analysis of 298 population-based surveys involving 1.6 million participants to assess global trends in insufficient PA among adolescents aged 11 to 17 years (8). Their findings indicated approximately 15% adherence in girls and around 22.5% in boys to PA recommendations worldwide, with noticeable variations across countries. In our study, we observed a slightly higher adherence to PA levels, ranging from 17.6 to 19.8% in girls and 27.7 to 31.1% in boys over the study period. However, our adherence rates appear somewhat lower than those reported by Steen-Johannsen et al. in their study on European children and adolescents, where two-thirds were identified as insufficiently physically active (9). It is essential to note that adherence to PA recommendations may decline from early to late adolescence, as evident in both our data and international findings (8). Therefore, caution should be exercised when comparing total average PA scores based on the average age across different populations and studies.

When comparing our findings of adherence to physical activity (PA) recommendations with earlier data from the UngKan3 study on 15-year-old Norwegian adolescents, we observed lower adherence rates than those reported by Steene-Johannessen and colleagues in 2018 (11). In their study, adherence rates of 40 to 50% were reported for Norwegian girls and boys, respectively. Despite the smaller sample size in the UngKan3 study, which included 1,325 participants from the 10th grade, it featured a comprehensive set of PA measures, including accelerometers, enhancing the validity of their findings. However, the response rate among 10th graders was 57.3%, introducing some uncertainty due to non-responders. In contrast, the larger nationwide sample in the Ungdata study provides valuable supplementary research evidence for mapping PA levels in Norwegian school-based adolescents, particularly over time. Surprisingly, our data showed no clear impact of the pandemic on PA engagement. This could be attributed to adolescents finding alternative ways to remain physically active or the rapid changes in restrictions during the pandemic, entailed respondents to complete the Ungdata survey while schools were open, and thus in a period of reduced restrictions.

Interestingly, our findings revealed a more pronounced decrease in adherence to PA recommendations among girls compared to boys from 8th to 3rd grade each year. The potential barriers explaining these gender differences are likely multifaceted, involving various factors. A recent systematic review by Martins and colleagues, examining adolescents’ perspectives on the barriers and facilitators of PA, identified five overarching themes: individual factors (e.g., self-efficacy), social and relational factors (e.g., friends and family), PA nature factors (e.g., school-based PA), life factors (e.g., time), and sociocultural and environmental factors (e.g., availability of PA facilities) (48). It is noteworthy that most studies included in this systematic review primarily recruited girls, particularly for the first two identified factors (individual and social/relational factors), which may play a more significant role in explaining gender differences compared to the other factors (PA nature, life factors) that are more structurally based. Additionally, findings among preadolescents highlighted that self-efficacy, rather than peer or parent support, was associated with higher PA and less sedentary time (49), emphasizing the importance of individual factors.

Self-efficacy emerges as one of the pivotal individual factors influencing physical activity (PA) engagement. The concept of self-efficacy is considered a self-regulatory mechanism capable of inducing change (31, 32). Promoting PA engagement to enhance self-efficacy appears to be a logical approach. According to Bandura, self-efficacy comprises several components (13): (i) Performance accomplishments (personal experience), (ii) Vicarious experiences (observations of others), (iii) Verbal persuasion (encouragement), and (iv) A person’s physiological state (physiological reactions). Intriguingly, engagement in PA naturally addresses all these components. Through PA involvement, adolescents not only augment their personal experience but also identify role models (observations of others), potentially increasing their belief in their own capacity. A meta-analysis by Ashford et al. explored the most effective strategies to change self-efficacy for promoting lifestyle and recreational physical activity in adults. Twenty-seven interventions were identified, demonstrating a significant relation to self-efficacy. Importantly, interventions reporting the most effective ways to promote self-efficacy included feedback on participants’ past performances (i), vicarious experiences (ii), and feedback by comparing performance (21). Thus, PA engagement appears crucial for promoting self-efficacy, providing feedback that adolescents can reflect upon. Additionally, coaches and schoolteachers, through facilitation and feedback, play an essential role in enhancing performance accomplishments and PA experiences in adolescents.

As hypothesized, adherence to PA recommendations (highest and lowest levels of PA) demonstrated a strong/inverse association with self-efficacy in girls and boys nearly every year from 2017 to 2021. The accumulated findings over the period provide solid evidence of the link between PA levels and self-efficacy among Norwegian adolescents. A positive feedback loop of PA engagement yields higher self-efficacy, further promoting and facilitating PA engagement. However, the bidirectional nature should be discussed, as correlates between PA levels and self-efficacy are well-known (50). In the study “Explaining adolescent exercise behavior change: a longitudinal application of the transtheoretical model” (51), a cross-lag panel design was employed to investigate the direction of the association between PA and self-efficacy. PA and self-efficacy were evaluated at baseline and after 3 years. Findings revealed that PA levels at baseline determined self-efficacy levels 3 years later, whereas self-efficacy did not predict PA levels 3 years later (51). However, other studies examining self-efficacy and PA using a cross-lag panel in other populations have reported self-efficacy as the determinant of the association (52, 53). Regardless of the directionality of the associations, PA levels and self-efficacy are interwoven phenomena that should be addressed in future observational and longitudinal research. Moreover, findings of low adherence to PA recommendations among Norwegian adolescents over time indicate the need for promoting PA on a structural level, such as implementing more mandatory PA in both lower and upper secondary school.

### 4.1 Strengths and limitations

This study boasts several strengths that contribute to its robustness. The utilization of nationwide data from all regions of Norway ensures a high level of representativity. The large number of participants and the consistently high response rate (ranging from 91.9 to 93.8%) regarding PA levels enhance the study’s validity. The incorporation of data from the Ungdata survey, acknowledged as the most comprehensive source of information on Norwegian adolescents’ health, further bolsters the study’s credibility. The dataset is meticulously cleaned, featuring stringent procedures for identifying unserious responses and a standardized, validated variable for socioeconomic status (SES) (33). Additionally, adherence to the STROBE guidelines (44) in reporting strengthens the study by ensuring accurate and consistent reporting practices. Finally, the accumulated consistency of the findings combined with the statistical strength throughout the study period using nationwide data, led to extensive evidence with clear differences over time, which enables a robust conclusion of findings.

While the annual data collection offers the advantage of assessing trends over time, it is crucial to recognize several inherent limitations. The anonymity of the data, without provided IDs for responders, prevents the implementation of repetitive statistical measures. Consequently, specific trends within the exact same study sample cannot be tracked. Municipalities have the flexibility to enter or exit the study, but a considerable portion tends to remain within the same study population each year, with 3rd graders discontinuing and new 8th graders entering. Descriptive stratification by grades and year proves useful in examining changes in specific cohorts over time.

A notable limitation stems from the lower sample size in the self-efficacy measure, attributed to a lower response rate and fewer municipalities including these questions. This reduction in sample size introduces risks of bias and diminishes the overall validity of the data. Furthermore, due to the comprehensive nature of health aspects covered in the Ungdata survey, questions often lack a clear origin (34). Therefore, a significant limitation lies in the use of a one-item non-validated instrument regarding PA level as a proxy for PA recommendations. The question exclusively gages the frequency of PA, neglecting other crucial aspects encompassed in PA recommendations, such as duration and intensity. Furthermore, bias related to gender differences in self-reporting of PA and self-efficacy might have influenced the validity of findings, as it is suggested that women tends to underestimate PA engagement and own performances compared to men (54, 55).

## 5 Conclusion

Our study, conducted among Norwegian adolescents from 2017 to 2021, uncovered significant gender disparities in adherence to physical activity (PA) recommendations and self-efficacy. Girls consistently reported lower adherence to PA recommendations and lower self-efficacy compared to boys during this period. Additionally, our data underscored robust associations between adherence to PA recommendations, levels of physical activity, and self-efficacy over time. These findings emphasize the crucial need for promoting adherence to PA recommendations in adolescence, with a specific focus on addressing the observed gender differences. The implications extend to policymakers and the Norwegian government, urging concerted efforts to create an environment conducive to increased physical activity among adolescents. By enhancing adherence to PA recommendations, policymakers can contribute not only to physical well-being but also to mental health, as reflected in improved self-efficacy. This, in turn, may empower adolescents to face and overcome challenges with a heightened belief in their own capabilities. The study advocates for targeted interventions and policies aimed at fostering a more active and resilient adolescent population in Norway.

## Data availability statement

The data analyzed in this study is subject to the following licenses/restrictions: Data supporting the results of this study is available upon request from the Norwegian Agency for Shared Services in Education and Research (SIKT). Reference to dataset from SIKT: https://doi.org/10.18712/NSD-NSD3007-V3. Requests to access these datasets should be directed to https://sikt.no/.

## Ethics statement

The studies involving human participants were reviewed and approved by Norwegian Agency for Shared Sevices in Education and Research (SIKT). The patients/participants provided their written informed consent to participate in this study.

## Author contributions

EG: Conceptualization, Data curation, Formal analysis, Funding acquisition, Investigation, Methodology, Project administration, Resources, Software, Supervision, Validation, Visualization, Writing – original draft, Writing – review & editing. ØS: Conceptualization, Investigation, Methodology, Supervision, Validation, Writing – original draft, Writing – review & editing.
