# Supplementary material for: Adherence to physical activity recommendations and associations with self-efficacy among Norwegian adolescents: trends from 2017 to 2021
Source: Front Public Health. 2024 May 23;12:1382028. doi: 10.3389/fpubh.2024.1382028 (PMC11155692; doi:10.3389/fpubh.2024.1382028)
Supplement: Supplementary file 1 [file Table_1.DOCX]

**Supplementary File 1**

Response rate in study variables

| Year | PA level  N | Response rate % | Self-efficacy*  N | Response rate % | Participating municipalities |
| --- | --- | --- | --- | --- | --- |
| 2017  2018  2019  2020  2021 | 100496  65638  111895  24449  130568 | 93.5%  91.9%  93.1%  93.8%  92.9% | 38260  6265  85186  12704  54371 | 76.1%  51.8%  77.4%  72.5%  65.3% | 26 out of 74  15 out of 48  72 out of 83  18 out of 29  50 out of 81 |

*Self-efficacy was not part of the mandatory Ungdata survey module, therefore a various number of participating municipalities each year.
